# Motion feature extraction based on semi-supervised learning and long short-term memory network in digital dance

**DOI:** 10.3389/fnbot.2026.1743288

**Published:** 2026-01-29

**Authors:** Xue Yang, Hanmin Sun, Yin Lyu, Yang Sun

**Affiliations:** 1 College of Music, Huaiyin Normal University, Huai'an, China; 2 Weihai Art College, Shandong University, Weihai, China; 3 Department of Dance School of Dance, Taipei National University of the Arts, Taipei City, Taiwan; 4 College of Artificial Intelligence, Shenyang Normal University, Shenyang, China

**Keywords:** CNN, linear regression, long short-term memory network, motion feature extraction, semi-supervised learning

## Abstract

Digital-image technology has broadened the creative space of dance, yet accurately capturing the semantic correspondence between low-level motion data and high-level dance key-points remains challenging, especially when labeled data are scarce. We aim to establish a lightweight, semi-supervised pipeline that can extract discriminative motion features from depth sequences and map them to 3-D key-points of dancers in real time. To achieve pixel-level alignment between dance movement targets and high-dimensional sensory data, we propose a novel LSTM-CNN (Long Short Term Memory-Convolutional Neural Network) framework. Temporal-context features are first extracted by LSTM, after which multi-dimensional spatial features are captured by three convolutional layers and one max-pooling layer; the fused representation is finally regressed to 3-D body key-points. To relieve class imbalance caused by complex postures, an online hard-example mining (OHEM) strategy together with a Dice-cross-entropy weighted loss (3:1) is embedded into semi-supervised learning, enabling the network to converge with only 20% labeled samples. Experiments on the public MSR-Action3D dataset (567 sequences, 20 actions) yielded an average recognition rate of 96.9%, surpassing the best comparison method (MSST) by 1.1%. On our self-established dataset (99 sequences, 11 actions) the accuracy reached 97.99% while training time was reduced by 35% compared with the previous best Multi_perspective_MHPCs approach. Both datasets show low RMSE (≤ 0.032) between predicted and ground-truth key-points, confirming spatial precision. The results demonstrate that the proposed model can reliably track subtle dance gestures under limited annotation, offering an efficient, low-cost solution for digital choreography, motion-style transfer and interactive stage performance.

## 1 Introduction

Since Cunningham first used the LifeForms software in 1989 to algorithmically generate limb sequences, digital tools had become integral to choreographic practice transforming the ephemeral body into computable motion data that could be stored, re-mixed and re-performed. This same lineage now drives the demand for real-time, marker-light extraction of 3-D dance key-points, yet the scarcity of annotated datasets remains a stubborn legacy of the studio’s ephemeral culture (Kalaiyarasi et al., 2024; Dos Santos et al., 2024).

Yet, once the performance is recorded, the question remains: how do we read the moving body? Screen dance scholars warn that cinematic tricks with slow-motion, whip-pans, upside-down shots may spectacularize motion without revealing its internal logic. To bridge the gap between visceral spectacle and analytical insight (Alqahtany and Syed, 2024), dance researchers have turned to motion capture, seeking numeric surrogates that preserve not only the trajectory but the “feel” of movement, what Laban called the Effort qualities of Weight, Time and Flow.

In the field of dance art, more and more choreographers have realized the advantages of computer software in dance application. For example, the great Merce Cunningham, using computer technology to develop a choreography software, through this computer software, it can store and edit the dance movements of each moment, because it is a virtual dance movement, so the choreographer can design any dance posture, find more body language. In the process of choreography, it can change the angle, adapt the speed, change the space at will, and get the better visual effect by constantly comparing. Not only that, the choreography software “Life composition” can randomly combine the joined body movements without human operation. This randomness will break the traditional dynamic connection and give birth to more novel shapes and dance movements (Dodd et al., 2024). As emphasized in reference (Jordan, 2020), AI’s value in artistic practice lied in its ability to translate abstract artistic intentions into computable logic while preserving the creative autonomy of artists, this perspective highlighted that effective digital dance technology should act as a collaborative tool, not a passive recorder. For dance, this meant motion feature extraction should not only capture geometric coordinates but also encode the artistic nuances (e.g., rhythmic flow, stylistic expression) that defined choreographic works, which aligned with our goal of bridging low-level motion data and high-level dance key-point semantics. As argued in Dance Becoming Knowledge (Leach and Delahunta, 2017), the digital transformation of dance was not merely a technical reproduction of physical movements, but a process of translating embodied dance knowledge (such as movement intention, stylistic norms, and expressive nuances) into computable forms. This perspective emphasized that effective motion feature extraction should go beyond capturing geometric coordinates, and instead encode the “know-how” inherent in dance practice, which aligned with our goal of bridging low-level motion data and high-level dance key-point semantics. Three domain-specific constraints shape our problem. (1) Ephemeral data: once the dancer leaves the studio, the moment is gone; any capture must be unobtrusive and immediate. (2) Esthetic validity: a 3 cm joint-error tolerable in gait-biometrics can shift a movement from “lyrical” to “robotic” in the eyes of a ballet master. (3) Annotation scarcity: Labeling every frame with 3-D key-points is labor-intensive, and dancers rarely sign the lengthy consent forms required for biometric datasets. Consequently, less than 20% of recorded material is ever annotated, a ratio that mocks the appetite of fully-supervised deep networks.

Dance, however, is not just another VR application. Its scholarly study demands concepts such as phrasing, weight and gaze that categories absent from surveillance-oriented motion datasets. Some dance researchers argue for movement-analysis tools that are legible to both algorithms and artists. We therefore frame the extraction of 3-D key-points not as a generic tracking problem but as the construction of a movement literacy layer that can be read by choreographers, annotated by dancers, and mined by scholars. The recognition of human motion video or image by computer vision technology is based on the analysis and processing of its video or image sequence. The motion feature extraction and classification recognition of the detected human moving objects are carried out to understand and describe their behavior. Human motion feature analysis based on video image has a broad application prospect in intelligent video surveillance, intelligent interface, virtual reality and other fields.

Following recent studies that treat kinematic dance sequences as behavioral biometrics (Jiang and Yin, 2023), it leverages movement signatures to distinguish among dancers. To mitigate the ethical risks, the present work adopts privacy-by-design principles (see Ethics Statement). The so-called biometrics technology, its specific operation is to use the biological characteristics inherent in the human body for individual identity authentication, the most significant feature is that it has invariance and uniqueness. Throughout this paper, four kinematic descriptors are employed as multi-dimensional inputs. (i) limb-swing angle: the maximum angular displacement between thigh and shank during one frame; (ii) gait cycle: time-normalized stride period extracted from heel-strike to heel-strike; (iii) torso symmetry: ratio of left- and right-shoulder vertical displacement; (iv) profile projection: width-to-height ratio of the dancer’s lateral silhouette. These features are selected for their invariance to camera viewpoint and costume color (Teng et al., 2023).”

Nowadays, there are many research algorithms in the field of motion feature recognition, including the establishment of space–time model of walking human body to extract motion features and characterize the appearance of walking human body. Image optical flow method is used to extract human motion features. The motion description of the image, that is, the general object structure, is used to acquire the characteristics of the moving human body in a certain way and then analyzed and processed to obtain its motion characteristics. At present, motion features in motion feature recognition include two kinds of components: structural- and dynamic- component. The structural component is the static component, which is responsible for recording the body shape information such as the height and stride length of the moving human body. The dynamic component vividly represents the motion characteristics of the human body during the movement, such as the angle of the arm swing and the way of the limb stride. According to the above two types of components, the existing motion feature recognition algorithms can be roughly divided into two categories: statistics-based method and model-based method (Xu et al., 2025).

The main method of using statistics-based method to obtain human motion features is to calculate some human motion parameters such as speed, contour, texture, etc. in continuous video image sequence, and further use its spatiotemporal statistical characteristics to classify and recognize. The statistical method usually assumes that the changes of human motion in the mode are highly consistent with the changes of pixels in the image sequence, and its motion feature parameters can be directly derived from the video image sequence, which greatly reduces the computational complexity and does not require the construction of simulation, so it is also called the non-modeling method. However, this method often results in motion features with high dimensionality, so it is necessary to apply the corresponding dimensionality reduction operation to the actual processing process.

In view of the above advantages, statistics-based method has become a popular feature recognition algorithm at present, and has a wide range of applications in motion feature recognition. In order to distinguish different modes of walking motion, Sulzbachner et al. (2011) proposed a time-flood-space correlation matching method. Tafazzoli and Safabakhsh (2010) proposed the theory that the head and feet had different movement patterns in the space–time dimension, and the edge features of human limb movement could be collected by processing these patterns. Liu (2016) introduced the generalized symmetry operator in motion feature recognition. Lam et al. (2007) proposed a baseline algorithm for gait recognition in motion. Sun et al. (2014) extracted the moving gait features represented by the outer profile of the human body in moving images, calculated the FED vector between each frame of a complete gait sequence and the sample set, and then used Hidden Markov Model (HMM) model to classify and identify moving targets.

Dance movements differ from general human movements and possess three core characteristics: rhythmic coherence, artisticity of movements, and standardization of postures. They must complete a coherent sequence of actions under a fixed rhythm, including the distinctive postures of specific dance styles (such as ballet and modern dance), and the changes in movement amplitude and joint angles follow artistic patterns. These characteristics impose higher requirements on the sequence capture ability and feature discrimination of the extraction model.

However, the shortcomings of the above methods are summarized as follows: (1) In the face of complex scenes, the details of the movement cannot be clearly distinguished, thus losing a large number of features; (2) When the motion state is more complicated, the features will be more redundant, thus reducing the recognition rate; (3) When deep learning networks are used, the convergence is relatively slow, which is not conducive to generalizing and solving other motion problems. (4) Dance motion data has inherent scarcity: due to the uniqueness of choreographic works, high cost of professional motion capture, and difficulty in standardizing artistic expressions, it is extremely challenging to obtain large-scale labeled training samples. This scarcity easily leads to overfitting of deep learning models, further limiting the accuracy and generalization ability of feature extraction. Therefore, this paper proposes a novel motion feature extraction model based on semi-supervised learning and long short-term memory network in digital dance. To address the limitations of traditional manual feature extraction (e.g., information loss and insufficient spatiotemporal feature capture), this study proposes a novel motion feature extraction framework integrating semi-supervised learning, LSTM, and CNN, with core contributions and detailed implementation logic as follows.

(1) Dual-branch feature extraction for spatiotemporal complementarity

We design a collaborative LSTM-CNN architecture to fully capture the dual characteristics of dance motions. LSTM is dedicated to modeling original multi-dimensional time-series motion data (e.g., limb position, movement velocity) collected by sensors. It effectively captures long-term dynamic dependencies in dance sequences, such as the order of limb movements, speed variation trends, and smooth transitions between action segments, avoiding the gradient vanishing problem of traditional recurrent networks. Meanwhile, CNN focuses on extracting multidimensional spatial features of dance motions, including the relative position of joints, limb angle relationships, and overall body contour structure, through multi-layer convolution and pooling operations that enhance spatial invariance and local feature sensitivity.

(2) Explicit feature fusion and targeted keypoint mapping

The temporal features extracted by LSTM (encoding dynamic motion logic) and spatial features extracted by CNN (encoding static structural information) are integrated through feature concatenation. This fusion strategy generates a comprehensive feature vector that simultaneously embodies the spatiotemporal attributes of dance motions, laying a foundation for accurate keypoint prediction. For the mapping from fused features to dance keypoints, we adopt a two-layer fully connected regression structure. The target keypoints cover 18 core body positions (e.g., head, shoulders, elbows, hips, knees) with 3D coordinates (derived from depth image data), and the regression process is optimized by a weighted loss function that balances class imbalance and pixel-level prediction accuracy, ensuring precise alignment between features and keypoint coordinates.

(3) Effective regularization to enhance model generalization

To mitigate over-fitting caused by limited motion data samples, we introduce the Dropout algorithm into the LSTM-CNN architecture. During training, a portion of neurons in the LSTM output layer and CNN fully connected layers are randomly masked, reducing interdependence between neurons and implicitly expanding the effective sample size. This technique enhances the model’s robustness to local feature noise and avoids over-reliance on specific motion patterns. During the test phase, Dropout is disabled, and neuron weights are adjusted to maintain consistent output distribution, ensuring the model’s adaptability to unseen dance motions.

Semi-supervised learning with hard example mining for performance improvement

Combining semi-supervised learning (SSSMKD) with online hard example mining (OHEM), we address the challenges of large morphological differences and unbalanced motion proportions in dance. The semi-supervised framework expands training samples through data enhancement and mutual knowledge distillation between teacher-student models, improving prediction confidence. OHEM screens difficult samples (e.g., motion segments with high prediction loss or ambiguous keypoints) and assigns higher weights to them during training, ensuring the model focuses on learning complex and error-prone motion features, thereby further enhancing extraction accuracy.

This paper is structured as follows to systematically present the research work. Section 2 provides a comprehensive review of related works in motion feature extraction, categorizing existing methods into feature-based and model-based approaches, and summarizing their advantages, limitations, and application scenarios. Section 3 elaborates on the detailed design and implementation of the proposed motion feature extraction framework, including background modeling, motion detection, LSTM-CNN joint feature extraction, semi-supervised learning with hard example mining, and regularization techniques. Section 4 presents the experimental setup, including the datasets (MSRAction3D and self-built database), parameter configurations, and evaluation metrics, followed by in-depth analysis of the experimental results to verify the effectiveness and superiority of the proposed method. Finally, Section 5 summarizes the main contributions and findings of this study, and outlines potential directions for future research.

## 2 Related works

To contextualize the proposed motion feature extraction model for digital dance, this section differentiates between general human motion analysis (focused on universal motion recognition) and dance-specific digital embodiment research (integrating artistic expression, choreographic logic, and bodily dynamics). We first review relevant literature in both domains, then articulate the research gap addressed by this study.

### 2.1 General human motion feature extraction

**Figure 1:**
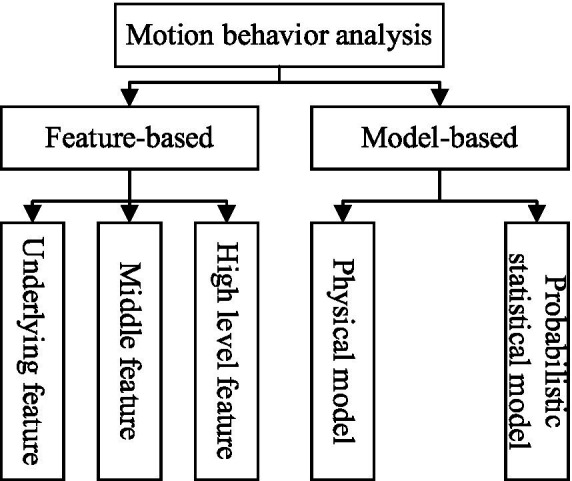
Classification of motion feature extraction method.

General motion analysis aims to recognize, track, and classify human movements across universal scenarios (e.g., walking, running, daily activities) and has formed two mainstream technical paradigms: feature-based and model-based methods (Zhu et al., 2025; Teng and Qiao, 2022) as shown in Figure 1.

(1) Feature-based Methods. These methods extract low-level visual features from video/image sequences and construct descriptors for classification. The Bag of Words (BoW) model encodes motion features into visual “words” to build behavior templates (Qader et al., 2019), but struggles with dynamic motion sequence continuity. Pawar and Attar (2019) extended the BoW model and used it to detect suspicious events in the video, assuming that the events with low frequency in the video were abnormal events. The method encoded video clips into compressed space–time bodies, which were modeled using probabilistic models considering the instability of constructing codebooks. If the input video clip did not build into the previously built descriptors, it was considered an exception. Manifold Learning captures local motion structures through spatial–temporal feature mapping, yet fails to handle high-dimensional motion data with redundant information. Ullah et al. (2022) applied the framework based on manifold learning to anomaly detection in crowd scenes, and used the Lagrange feature mapping method with spatial–temporal characteristics to learn the local motion structure of crowd scenes. Stability analysis identifies crowd motion patterns via particle trajectory and Jacobian matrix stability, but is limited to macroscopic group behavior and cannot capture fine-grained individual motion details. Matkovic et al. (2022) introduced the concepts of regions of interest (ROI) and Eigenvalue maps for crowd scenes. The trajectories of moving particles in crowded scene were obtained by time integration of dynamic system. These trajectories were used to locate ROI in the scene. The Jacobian matrix was introduced for linear approximation to the dynamic system, and the stability of the particle in the local region was identified according to the parameters of the matrix (the rank and trace of the matrix), so as to identify five types of behaviors in the video: bottlenecks, fountainheads, lanes, arches and blocking.

Bottlenecks: Refers to narrow spatial regions where crowd flow is constrained. Particles (representing individuals) show concentrated trajectories with reduced speed, and the direction of motion tends to be consistent (e.g., crowds passing through a narrow door or corridor). The Jacobian matrix of such regions shows low stability due to the compression of motion space.

Fountainheads: Describes the source regions of crowd diffusion. Particles start from a centralized location and spread outward in multiple directions, with trajectories showing radial distribution (e.g., crowds dispersing from a stage or a central gathering point). The local motion stability in these regions is high, and the particle velocity gradually increases as they move away from the center.

Lanes: Denotes the orderly, linear motion paths formed by crowds. Particles move along parallel or nearly parallel trajectories with consistent direction and stable speed (e.g., pedestrians walking in orderly lanes on a sidewalk or corridor). The Jacobian matrix parameters of lane regions show uniform stability, reflecting the regularity of crowd motion.

Arches: Refers to arc-shaped motion trajectories formed by crowds avoiding obstacles or following curved spatial constraints. Particles move along a smooth arc path, with the direction of motion gradually changing but maintaining continuity (e.g., crowds bypassing a circular obstacle or moving along a curved corridor). The local stability of such regions fluctuates slightly with the curvature of the trajectory but remains within a stable range.

Blocking: Represents the state where crowd motion is stagnant or severely hindered. Particles show almost no displacement, and trajectories are concentrated in a fixed region (e.g., crowds being blocked by a sudden obstacle or a stampede). The Jacobian matrix of blocking regions shows extremely low stability, indicating the loss of normal motion dynamics.

(2) Model-based Methods. These methods fit motion dynamics using prior models. The Floor Field Model (FFM) analyzes high-density crowd flow by simulating “local/global force” effects, but is ineffective for discrete, artfully designed dance movements. Abdullah and Jalal (2023) successfully used FFM for motion pattern segmentation and anomaly detection in crowd scenes. Hameed et al. (2021) proposed a new model. In this model, when the target individual moves in a specific scene, it would be affected by a local or global force. This force was a function of the scene layout and the individual behavior leading the crowd to move, in other words, the normal flow of the crowd and the constraints of the scene would affect the individual behavior in the crowd, and this behavior had a certain role in predicting the direction of the individual and tracking the target in advance. The Random Field Topic (RFT) model clusters trajectory fragments to learn semantic regions, yet lacks adaptability to non-linear, expressive dance motion transitions. For moving targets in crowd scenes, Zou et al. (2014) proposed RFT to learn semantic regions of trajectory fragments. At the same time, the popular latent dirichlet allocation topic model was also used to integrate Markov random fields to strengthen the spatio-temporal correlation between trajectory fragments during the learning process. And the random forest spanning tree was used to analyze the trajectory fragments.

While general methods achieve good performance in daily motion recognition, they face inherent limitations when applied to dance: (1) Ignoring the artistic continuity of dance—dance movements are not isolated actions but choreographically designed sequences with emotional and rhythmic logic; (2) Failing to handle dance-specific motion characteristics (e.g., large limb swings, complex body postures, and subtle muscle contractions); (3) Requiring large-scale labeled data, which is scarce in dance due to the uniqueness of choreographic works.

### 2.2 Dance-specific digital embodiment research

A comprehensive survey (Preciado-Azanza and Akinleye, 2020) systematically summarized the integration of digital technologies into choreographic practice, pointing out three core trends: (1) digital capture of dance movements for preservation and reuse; (2) AI-assisted generation of choreographic sequences; (3) interactive feedback between dancers and digital media. The survey also emphasized two key challenges in current research. First, the lack of tools that could accurately map low-level motion data to high-level choreographic semantics (e.g., stylistic expression, rhythmic coherence); second, the over-reliance on large-scale labeled data in deep learning methods, which was inconsistent with the scarcity of dance motion annotations in practical choreographic scenarios. These conclusions aligned with the research gaps identified in this study, highlighting the necessity of developing lightweight, semi-supervised motion feature extraction models that aligned with choreographic needs. More recently, Nogueira et al. (2024) conducted a systematic review on the impact of machine learning on dance performance, covering 60 + studies and confirming that the core bottlenecks of current research lied in “insufficient integration of artistic expression” and “over-reliance on labeled data,” this review updated the research landscape of dance digitalization, further validating the necessity of our semi-supervised, art-aligned motion feature extraction model.

Dance-specific digital research integrates computer technology with choreography and dance studies, focusing on digital capture, analysis, and creative reuse of dance movements. This field has evolved around three core directions:

(1) Dance Motion Capture and Feature Representation

Early digital dance research focused on motion capture to preserve and reproduce dance movements. For example, Cunningham’s “Life Forms” (the first choreographic software) used 3D motion modeling to generate virtual dance postures, but relied on manual editing and lacked automatic feature extraction (Cunningham and Scharper, 2014). Later, Ya (2025) used depth data to optimize human pose estimation for dancers with upper limb impairments, verifying the feasibility of depth images in dance motion analysis, but their model only focused on pose correction and not on motion sequence feature fusion.

Recent studies have explored dance-specific feature representation. Chen et al. (2016) proposed depth motion maps (DMMs) for action recognition, but their method ignored the temporal dependencies between dance movements. Martin (2008) further emphasized that dance-specific features were not limited to spatial–temporal parameters but included stylistic and expressive nuances; for example, the tension in a limb or the fluidity of a transition that defined a choreographer’s unique language. Alohali et al. (2025) designed a smart activity recognition system for disabled people, which has reference value for capturing dance movements with special postures, but failed to address the class imbalance of dance motion samples (e.g., rare choreographic gestures). DeLahunta (2017) took Wayne McGregor’s “Choreographic Language Agent” as a case study, illustrating how AI could encode choreographic language (e.g., movement logic, stylistic norms) into computable agents, this work verified that AI-assisted choreography should not only focus on motion synthesis but also on the preservation and reuse of choreographic language, which aligned with our model’s goal of bridging low-level motion data and high-level choreographic semantics. Jones and Arts and Culture Google (2019) launched the “Body, Movement, Language” experiment in collaboration with Google Arts & Culture, which integrated AI, choreography, and natural language processing, this project allowed dancers to input textual descriptions of movement (e.g., “lyrical arm swing”) and generate corresponding choreographic sequences, demonstrating that AI could bridge the gap between abstract artistic language and concrete motion expression. This practice further supported that AI-assisted choreography should prioritize “human-machine collaboration” rather than full automation, which aligned with our model’s design principle of serving as a collaborative tool for choreographers.

(2) Computational Choreography and AI-Assisted Creation

Computational choreography aims to use algorithms to simulate or generate choreographic sequences. Pascoli et al. (2025) introduced agent personality into crowd simulation for immersive VR dance performances, enhancing the realism of virtual dancers but lacking alignment with professional choreographic rules. Shi and Huang 2026 applied Bayesian optimization and Transformer models to tennis motion recognition, providing a reference for sports motion sequence analysis, but dance motion differs from sports in its emphasis on artistic expression over functional efficiency, requiring models that capture both dynamics and expressiveness. Nogueira et al. (2023) further explored the integration of machine learning and dance visual expression by training models to interpret dance motion features and map them to novel visual representations, it demonstrated that technical extraction of motion features could directly serve artistic perception innovation. This study expands the application scenario of dance motion feature extraction from pure recognition to visual creation, highlighting the necessity of capturing both physical dynamics and artistic semantics in feature extraction, which aligns with our model’s design goal of mapping low-level data to high-level dance key-points with artistic significance. Chan et al. (2019) further advanced dance motion generation by proposing a pose-transfer framework. It extracted human poses from reference dance videos and mapped them to arbitrary target images, enabling realistic dance motion synthesis. This study demonstrated the critical role of accurate motion feature extraction in downstream choreographic applications (e.g., motion-style transfer), but its reliance on fully supervised training with large-scale labeled pose data limits its adaptability to dance scenarios, where annotated datasets were scarce due to artistic uniqueness and capture costs. This limitation aligns with the technical gap addressed in our work: developing a semi-supervised framework that reduces annotation dependence while preserving motion feature discriminability for dance-specific applications.

(3) Dance Motion Analysis for Multimodal Communication

Digital dance emphasizes the interaction between body movement and digital media, requiring motion features to reflect both physical dynamics and artistic meaning. Zhang et al. (2009) used trajectory clustering to analyze motion semantics, but their research focused on crowd behavior and not on dance’s symbolic motion language. Teng and Qiao (2022) proposed a context attention model for image semantic segmentation, which can extract spatial features of dance postures but ignores the temporal continuity of dance sequences. Correia et al. (2022) further explored the interactive visual design of dance based on body maps, proposing a composite animation method integrated with machine learning, this approach mapped high-dimensional body motion data to interactive visual elements in real time, verifying that motion feature extraction could directly support the innovation of dance digital visuals, which supplemented the technical gap of “motion-data to visual-expression mapping” in current multimodal communication research.

### 2.3 Research gap

Synthesizing the above literature, existing research has two critical gaps that this study addresses:

- Domain Adaptation Gap: General motion analysis methods ignore dance’s artistic continuity and unique motion characteristics (e.g., rhythmic transitions, expressive postures), while existing dance-specific research focuses on motion capture or virtual performance but lacks a unified framework for extracting spatiotemporal features that integrate physical dynamics and choreographic logic.
- Technical Limitation Gap: Current dance motion extraction methods either rely on manual feature engineering (losing fine-grained information) or use single-modal feature extraction (e.g., only spatial or temporal features), leading to insufficient feature representation. Additionally, labeled dance motion data is scarce, and semi-supervised learning has not been fully applied to address this scarcity.
- Application Alignment Gap: Few studies link motion feature extraction to practical digital dance applications (e.g., choreographic reuse, motion retrieval, or AI-assisted creation). Most methods focus on recognition accuracy rather than the usability of extracted features for artistic practice.

To fill these gaps, this study proposes a semi-supervised learning-based LSTM-CNN framework that: (1) Captures dance’s spatiotemporal continuity via LSTM (temporal) and CNN (spatial) joint extraction; (2) Uses semi-supervised learning with hard example mining to adapt to scarce dance data; (3) Maps features to dance keypoints with artistic and physical significance, supporting downstream digital dance applications.

## 3 Proposed motion feature extraction

**Figure 2:**
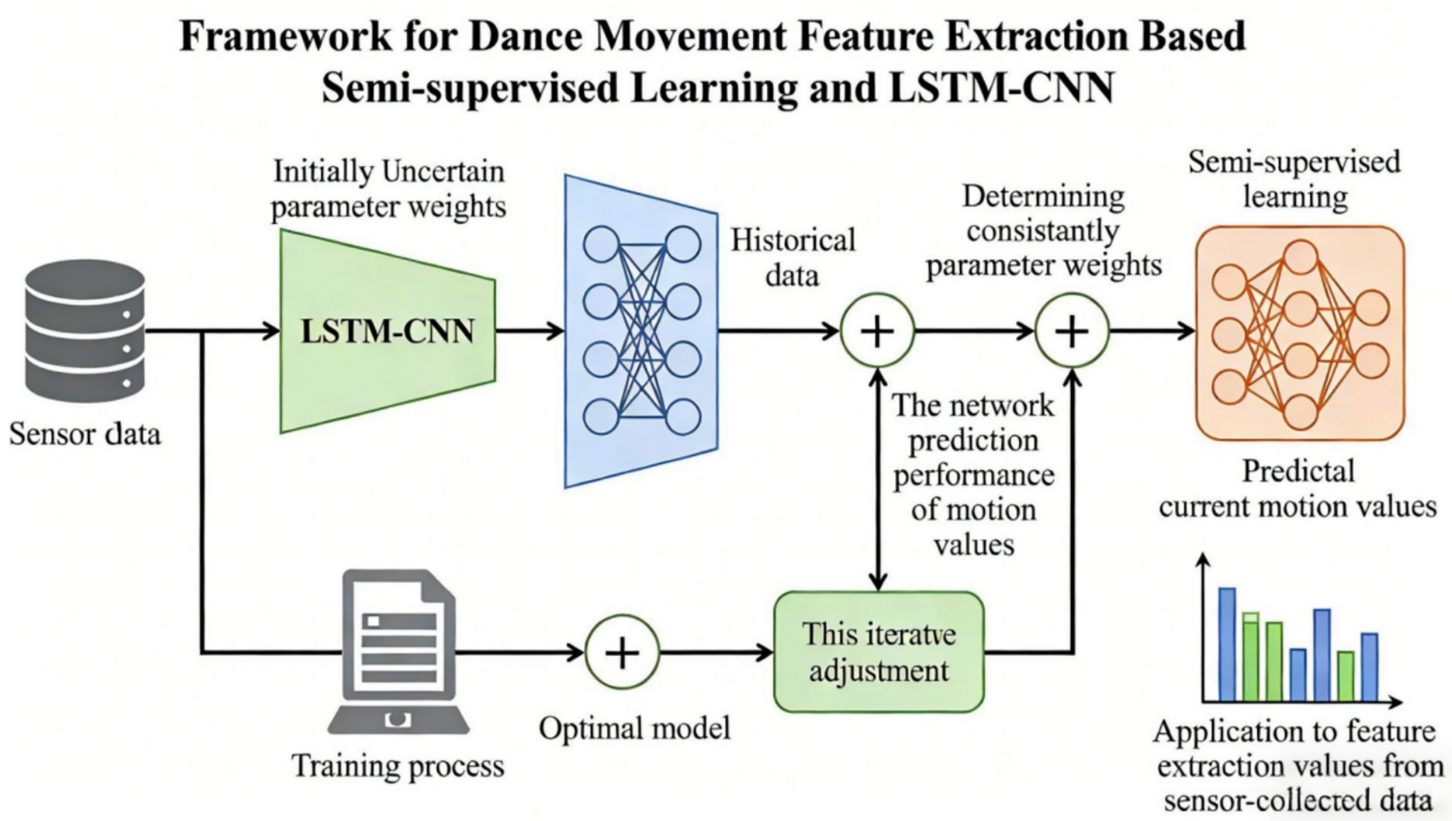
Proposed method.

The framework of dance movement feature extraction based on semi-supervised learning and LSTM-CNN is shown in Figure 2. The parameter weights of the LSTM-CNN network remain undetermined initially, so historical motion data is utilized to train the network and thereby determine the optimal parameter weights. According to the prediction performance of the network to the motion value, the weights of the parameters in the network are adjusted constantly, and finally an optimal model is obtained. The optimal model based on semi-supervised learning is applied to feature extraction to predict the current motion value according to the current data collected by the sensor.

### 3.1 Background modeling

Constructing a proper background model is the first step of using background subtraction method to detect moving objects. Common background modeling methods include mixed Gaussian model method, mean value method and median method. The mean value method and the mean value method are suitable for the case of strong robustness to the change of scene, and the principle is relatively simple and easy to be implemented.

The Least Median of Squares (LMEDS; Pascoli et al., 2025) is used to establish the background model of moving video images. The principle of background modeling with median method is that the time interval of shooting each frame of walking motion image is very short, the image content does not change much, and the pixel value of several frames of images at the same position is recorded. The pixel value of the background image at this position can be expressed as the middle pixel value, so that a more accurate background image can be obtained. The specific operation process is as follows. Firstly, it is assumed that there is a walking motion video sequence composed of (1,2,⋯,n) frames. Then the gray value of the point at the same position as (x,y) in this n frame image can be collected to obtain an array sequence $P_{i}(x,y),i=1,2,\cdots ,n$. Where i indicates the number of image frames. Therefore, the pixel value of the background image corresponding to the point can be expressed by the middle value of the sequence of pixel values of the n-frame image, as shown in Equation 1:


B(x,y)=Median(P(x,y)) (1)

Where B(x,y) is the pixel value of the background image at the point (x,y). (x,y) represents the position of the pixel.

### 3.2 Motion detection

Motion detection is the detection of moving objects in the original image. The precise extraction of motion region is helpful to improve the recognition effect of motion features to some extent. At present, several popular motion detection methods are inter-frame difference method, optical flow method and background subtraction method (Islam and Ali Khan, 2024). Because the method of inter-frame difference method is based on the difference and thresholding of pixels to extract the moving region of contiguity images of multiple frames, it is easy to have holes in the detection results, and generally only boundary points can be used to depict moving targets. However, the optical flow method takes the characteristics of optical flow changing with time as the basis to detect moving objects. Although it has strong robustness to the changes of shooting scenes, the calculation is complicated, the real-time performance and practicability are poor, and the accuracy of the extraction results of moving human bodies is low. At present, the most commonly used method in motion detection is background subtraction, which is realized by differential operation between the background frame and the current frame. In static background, the moving object extraction effect is better.

### 3.3 LSTM for model training

The original time series data $X_{k}$ collected by the sensor is used as the data input of the LSTM-CNN network. The input data is standardized as input to the LSTM neural network. LSTM neural network is used to model the standardized sequence data and extract the sequence features, and the output at the last moment in LSTM neural network is used as the input of CNN (Srinivas and Ganivada, 2025). Three convolution layers and one pooling layer are used to extract multi-dimensional features. The final output feature contains both the multi-dimensional feature and the sequence feature of the original time series data, and the feature is mapped to the motion value through the regression layer.

The input data $X_{k}$ of LSTM-CNN is the time series data collected at the k−th action state in the process of motion. $X_{k}=(X_{k}^{1},X_{k}^{2},\cdots ,X_{k}^{T})$, $X_{k}\in R^{1\times d}$. $X_{k}$ is a tensor of the shape 1×d, representing the multidimensional sensing data collected by the sensor at time t. d is the dimension of data collected by the sensor. T is the length of the sequence data. We set d=7 and T=100. In order to improve the generalization ability of the network, the sequence data $X_{k}$ is normalized and input to the LSTM neural network for sequence modeling and extraction of sequence features. The time step in the LSTM neural network is set to 100. The input data dimension is set to 7 at each moment, and the number of hidden layer neurons is 64. Therefore, the number of sequence features output by the hidden layer at the end point of LSTM neural network is 64. The hidden layer of LSTM neural network outputs $h_{T}$ at the end time T as the input of CNN. Multi-dimensional feature extraction is carried out by three convolutions and one maximum pooling operation in CNN. The convolution kernel size of the 3-order convolution is (3,3), the step size is 1, the convolution depth is 8, 16 and 64, respectively, and the number of output features is 1,024. Finally, the mapping to the motion eigenvalue $y_{\mathrm{pre}}$ is realized in the regression layer by twice fully connection.

In LSTM-CNN, in order to avoid the network convergence caused by abnormal data in the sample data and improve the learning speed and generalization ability of the network, it is necessary to standardize the original data. Z-score method is used to standardize the original data. Z-score is standardized as follows as shown in Equation 2.


$$Z=\frac{x-\mu }{\sigma_{0}}$$ (2)

Where x is the original data. μ is the mean of the original data. $\sigma_{0}$ is the variance of the original data. Z is the data after standardized processing, and Z is used as the direct input of the LSTM neural network.

The normalized sequence data is input into the LSTM neural network for sequence modeling and sequence feature extraction. Unlike traditional RNNs, LSTM alleviates the gradient exploding problem by introducing three core gates (forget gate $f_{t}$, input gate $i_{t}$, output gate $O_{t}$ and a cell state $C_{t}$), enabling accurate capture of long-term dependencies in dance motion sequences. The update process of the gate states, cell state, and hidden layer state follows a strict logical order, with specific equations and explanations as follows.

(1) Forget gate calculation

The forget gate determines how much historical information from the previous cell state $C_{t-1}$ is retained. It outputs a value between 0 and 1 through the sigmoid activation function σ. Its calculated as Equation 3.

$$f_{t}=\sigma (W_{f}(h_{t-1},X_{k}^{t})+b_{f})$$ (3)

$W_{f}$ is the weight matrix of the forget gate, learned during model training. $h_{t-1}$ is the hidden layer state of the LSTM at the previous time step t−1, encoding historical motion sequence features. $X_{k}^{t}$ is the input motion data at the current time step t (7-dimensional sensing data). $b_{f}$ is the Bias term of the forget gate, learned during training.

(2) Input gate calculation

The input gate controls how much current time step information is stored in the cell state $C_{t}$, which also relies on the sigmoid activation function as shown in Equation 4.


$$i_{t}=\sigma (W_{i}(h_{t-1},X_{k}^{t})+b_{i})$$ (4)

$W_{i}$ is the weight matrix of the input gate, learned during training. $b_{i}$ is the Bias term of the input gate, learned during training.

(3) Candidate cell state calculation

Based on the input gate’s decision, a candidate cell state ${\tilde{C}}_{t}$ is generated to record new motion information, using the tanh activation function to map values to the range [−1,1] as shown in Equation 5.


$${\tilde{C}}_{t}=\tanh(W_{C}(h_{t-1},X_{k}^{t})+b_{C})$$ (5)

$W_{C}$ is the Weight matrix of the candidate cell state, learned during training. $b_{C}$ is the Bias term of the candidate cell state, learned during training. ${\tilde{C}}_{t}$ is the candidate cell state, containing new motion features extracted at the current time step.

(4) Cell state update

The final cell state $C_{t}$ is updated by fusing the retained historical information (from the forget gate) and the new information (from the input gate and candidate cell state), using element-wise multiplication ⊙. Its expression is as shown in Equation 6.


$$C_{t}=f_{t}⊙C_{t-1}+i_{t}⊙{\tilde{C}}_{t}$$ (6)

(5) Output gate calculation

The output gate determines which information in the current cell state $C_{t}$ is output to the hidden layer state $h_{t}$, using the sigmoid activation function. Its expression is as shown in Equation 7.


$$O_{t}=\sigma (W_{O}(h_{t-1},X_{k}^{t})+b_{O})$$ (7)

$W_{O}$ is the weight matrix of the output gate, learned during training. $b_{O}$ is the Bias term of the output gate, learned during training.

(6) Hidden layer state update

The hidden layer state $h_{t}$ (which serves as the sequence feature output of the LSTM at time t) is obtained by activating the updated cell state $C_{t}$ with tanh and multiplying it by the output gate’s output. Its expression is as shown in Equation 8.


$$h_{t}=O_{t}⊙\tanh(C_{t})$$ (8)

At each time t, the cell state $C_{t}$ is updated by the input gate $i_{t}$, the forget gate $f_{t}$, the output gate $O_{t}$, and the hidden layer state $h_{t-1}$ and the cell state $C_{t-1}$ of the previous moment. The hidden layer state $h_{t}$ in the cell is updated based on the data entered at the current moment and the cell state $C_{t}$. Parameters $W_{f}$, $W_{i}$, $W_{C}$, $W_{O}$ and $b_{f}$, $b_{i}$, $b_{C}$, $b_{O}$ in the above formula are learned through model training and are shared by all times. n is the number of hidden layer neurons. T is the time step. ⊙ stands for element-by-element product. σ() indicates the sigmoid activation function. tanh represents the tanh activation function.

**Figure 3:**
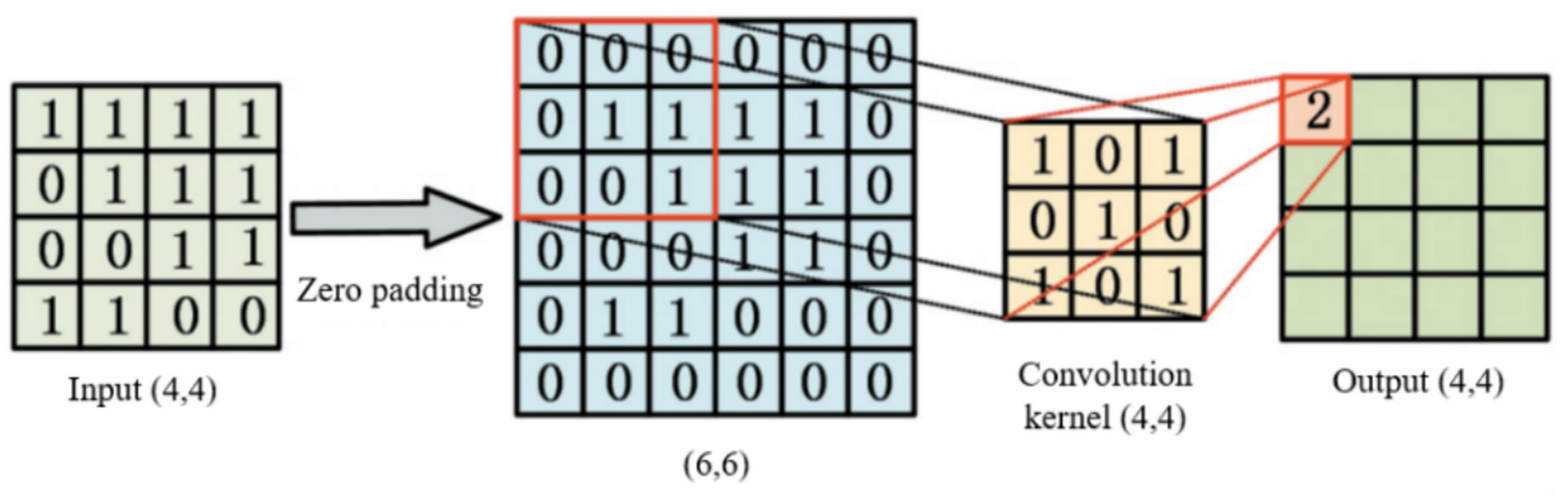
Convolution process.

The output $h_{T}$ of LSTM neural network at the end point is taken as the input of CNN. The input of CNN is successively extracted by convolution 1, maximum pooling, convolution 2 and convolution 3. The convolution process is shown in Figure 3 and the Equation 9:


$$A_{i,j}=f(\sum_{m=0}^{3}\sum_{n=0}^{3}w_{m,n}h_{i+m,j+n}+b)$$ (9)

Where w is the convolution kernel, whose shape is a two-dimensional matrix. $w_{m,n}$ is the element value of the m−th row and n−th column in the convolution kernel matrix. $h_{T}$ is the convolution layer input with matrix form. $h_{i+m,j+n}$ represents the element values of row i+n−th and column j+m−th in the convolutional layer input matrix $h_{T}$. b is the offset term. f is the Relu activation function.

Due to the high complexity of deep learning models, large-scale training data is critical to the robustness of the models. However, in motion feature extraction, it is difficult to obtain large-scale training data samples, and too small data samples for model training may easily lead to model over-fitting. Therefore, it is necessary to regularize LSTM-CNN to alleviate the phenomenon of model over-fitting. We introduce Dropout algorithm into the LSTM-CNN model to implement regularization of the LSTM-CNN model. The algorithm randomly blocks a part of neurons in each model training, which makes the network structure change slightly and forms a new network structure. Therefore, for the same input sample, it is equivalent to training on different neural networks, which reduces the dependence between neurons, that is, each neuron does not depend on several other neurons, so as to achieve the effect of expanding the sample size of data. In addition, the random shielding of neurons in the training process reduces the sensitivity of the neural network to local features in the process of feature extraction, so that the neural network can learn more robust features with other neurons, thus improving the robustness of the network. During the training process of the neural network after adding the Dropout algorithm, some neurons in the neural network will be randomly shielded to form a new neural network structure. The entire forward propagation process from the input of data samples to the output of prediction results is conducted in the new network structure. Therefore, the neurons that are randomly shielded will not affect the forward propagation process of the neural network. After the model training is complete, during the test phase, the Dropout will be turned off and the output of all hidden neurons will have an impact on the model test. For the LSTM-CNN model, adding Dropout to the LSTM neural network means that the neurons in the output layer of the LSTM network will be randomly screened during the training phase, that is, the sequence features extracted by the LSTM neural network after the data sample is input. Some sequence features will be randomly discarded and then input into CNN to continue multi-dimensional feature extraction.

### 3.4 Semi-supervised learning framework with online hard example mining for dance motion feature extraction

Semi-supervised semantic segmentation with mutual knowledge distillation (SSSMKD) is a semi-supervised learning model based on the consistent regularization method. This model combines the advantages of Mean-teacher model (Shi and Huang, 2026) and Cross pseudo supervision (CPS). By constructing two Mean-teacher models, the image input of the teacher model is weakly enhanced to improve the confidence of the prediction. The image input of the student model is strongly enhanced to expand the training sample, the pseudo-annotation generated by one teacher is used to supervise another student network, the parameters of the student model are updated by exponential moving average (EMA; Yu et al., n.d.) to get a new teacher model, and the other group is also updated. The process can be iterated to achieve the mutual knowledge distillation between the two branches. In addition, consistency constraints were applied to the output of the student models of the two branches, that is, the prediction results of the same image in the two Mean-teacher models should be consistent.

There are many inherent characteristics in dance movements that challenge feature extraction accuracy, such as significant morphological differences between different choreographic actions and imbalanced proportion of movement categories in training samples. The former refers to the large variations in body postures, limb swing ranges, and movement rhythms across dance styles (e.g., the gentle fluidity of contemporary dance vs. the intense dynamism of hip-hop); the latter means that individual movement types (e.g., basic steps vs. complex acrobatic movements) have highly uneven sample sizes in the training dataset. Such imbalances and differences lead to the model overfitting to high-frequency, simple movements while failing to learn sufficient features from low-frequency, complex ones, thereby reducing overall extraction accuracy. However, the accuracy of model extraction can be effectively improved by mining difficult to identify target regions and updating model parameters for difficult samples. Online hard example mining (OHEM; Ahn and Cutkosky, 2024) was first proposed and applied in the field of target detection, in which the model determined candidate frames of the target of interest and completed classification and positioning correction. The difficult samples are the feature maps in candidate frames with high losses, large candidate frame offset and unclear categories. Different from the traditional OHEM that focuses on candidate box screening in target detection, this study proposes an image-level pixel-wise difficult sample screening mechanism tailored to dance motion feature extraction. Specifically, the entire input depth image (containing complete dance motion information) is treated as an integral sample. After forward propagation through the LSTM-CNN network, pixel-wise prediction results and corresponding loss values (calculated based on the weighted loss function $L_{W\text{eighted}}=3L_{D\mathrm{ice}}+L_{CE}$) are generated for each pixel in the image. Subsequently, statistical aggregation (adopting the mean loss calculation method) is performed on the pixel-wise losses to obtain the overall loss of the image. Difficult samples are identified through a two-step threshold-based screening: first, samples with a confidence level (derived from the inverse of the normalized loss value) lower than the preset threshold p=0.7 difficult samples is less than the minimum pixel threshold D=100000, low-confidence simple samples are sequentially selected and adjusted to difficult samples until the pixel quantity requirement is met.

This screening mechanism highlights two key innovations for digital dance research: (1) It abandons the traditional candidate box-based local screening paradigm and adopts image-level integral screening, which is consistent with the global continuity of dance motions (dance actions involve large-range limb movements that cannot be split into local candidate boxes); (2) It integrates pixel-wise loss calculation with statistical aggregation, enabling precise capture of difficult-to-extract regions in dance motions (e.g., high-dynamic joint positions, subtle muscle contractions) that are easily ignored by local screening methods.

**Figure 4:**
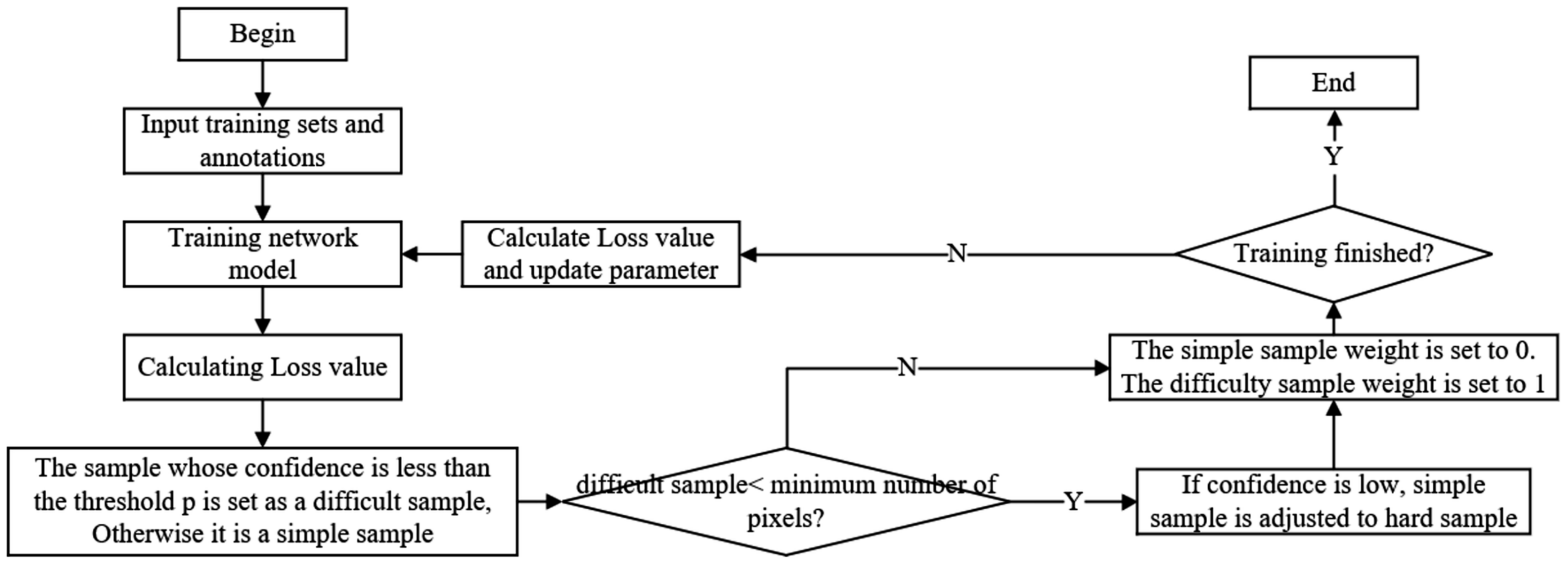
OHEM strategy.

The minimum number of pixels D=100000 and confidence p=0.7 are selected as the threshold values of the difficult pixel samples. When the original image is input to the network, the confidence p of each sample is calculated with the Loss value. The higher Loss value denotes the smaller confidence p. All samples with a confidence level less than 0.7 are considered difficult samples. If the total number of pixels of all difficult samples is less than the set minimum number of pixels D, the simple samples with low confidence are ordered from low to high according to the confidence of simple samples, and the simple samples with low confidence are adjusted to difficult samples to ensure that there are enough pixels in the training rounds to participate in training, thus improving training efficiency. The image containing the maximum loss of D pixels or all images with confidence less than p is set as difficult samples, and then the loss value of simple pixel samples is zeroed for subsequent gradient calculation and network update, and finally a round of online difficult sample mining strategy model training is realized. Figure 4 is a flow chart of the OHEM strategy. Regarding the ambiguity issue of high-difficulty transitional movements in dance (such as rotation-jump transitions), the OHEM strategy focuses on extracting specific difficult examples of dance movements to enhance the model’s accuracy in recognizing the distinctive dance actions.

The common cross entropy loss function (LCE) is difficult to deal with complex motions, because the proportion of motion samples in the training set is not balanced, resulting in a small value of the cross entropy loss function (LCE), which is not easy to help the model reach the optimal. However, Dice Loss is not affected by the image size, and only determines the segmentation effect by the intersection of two regions. It can help cross entropy loss function LCE to obtain better optimization effect. Therefore, Dice Loss can be used to measure the difference between the segmentation result and the real value from the target region to solve the class imbalance problem. At the same time, the cross entropy loss function LCE is used to measure the difference between the target and the real value at the pixel level. The adopted Loss function is the weighted superposition loss function (the weight ratio of Dice Loss and cross entropy loss function LCE is 3:1). Weighted loss function L-Weighted loss function, Dice loss function L-Dice and cross entropy loss function LCE are calculated as Equations 10–12:


L−Weighted=3L−Dice+LCE (10)

$$L-\text{Dice}=1-\frac{2|X\cap Y|+1}{|X|+|Y|+1}$$ (11)

$$\mathrm{LCE}=-\sum_{i=1}^{N}y_{i}\log p_{y\prime_{i}}$$ (12)

Where X and Y represent labels and prediction results, respectively. ∣X∣ and ∣Y∣ indicate the sizes of X and Y. ∣X∩Y∣ indicates the intersection size of X and Y.

**Figure 5:**
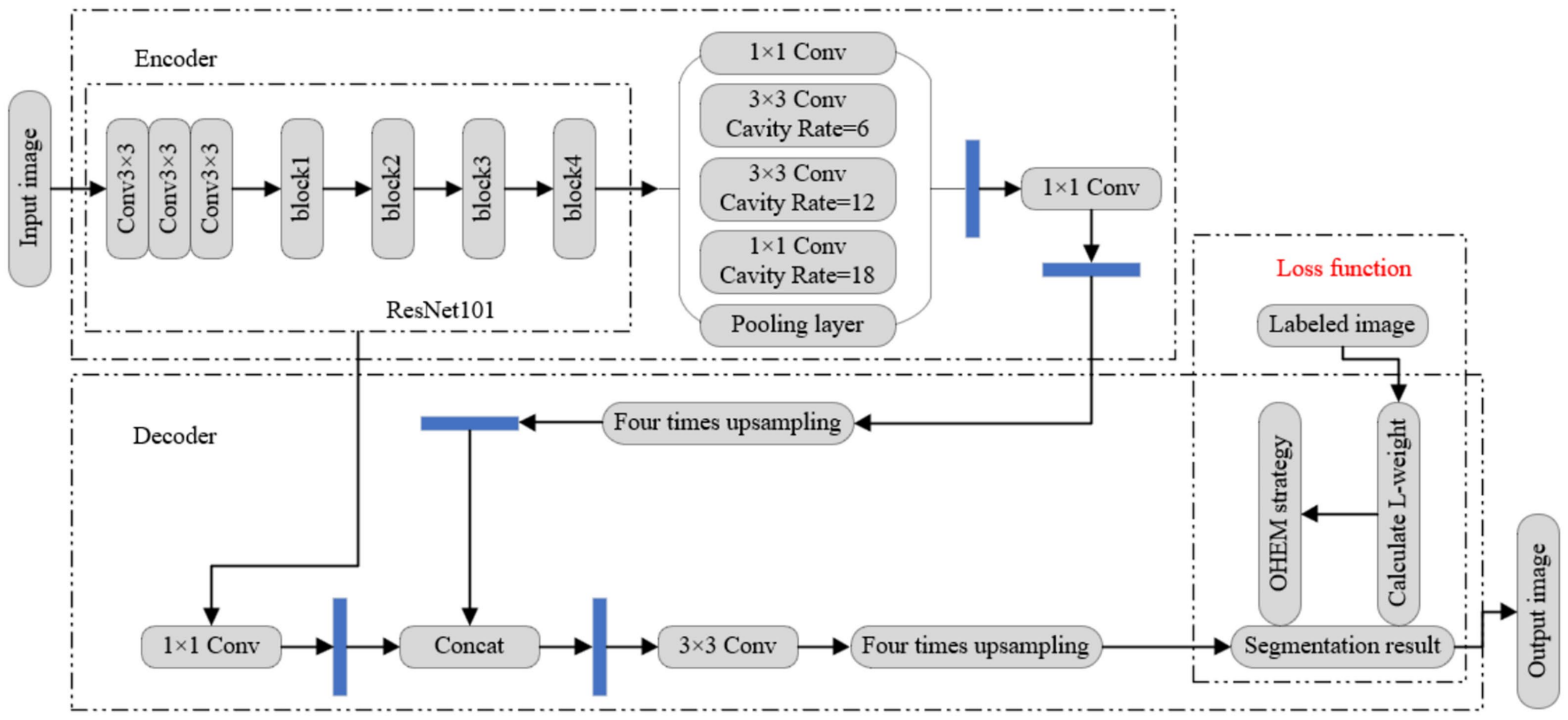
Improved DeepLabv3 + network.

To enhance the backbone feature extraction capability of DeepLabv3+, an improved ResNet101 is integrated into the network as the foundational feature extraction module. The structure of DeepLabv3 + model is shown in Figure 5. Its encoder is composed of backbone network and Atrous spatial pyramid pooling (ASPP) module (Salavati et al., 2025). At the encoder end, ResNet101 residual network model is used as the backbone network to perform 16-fold down-sampling to obtain the deep-level feature extraction map, and the multi-scale feature map can be obtained through ASPP module. At the decoder end, the multi-scale feature maps are obtained by connecting four time up-sampling and 1×1 convolution processing. Concat is performed on the processed feature graphs. Then the feature map of the original image size can be obtained by convolution and four times up-sampling, and the loss function is calculated here, and the network parameters are iterated accordingly. The final semantic segmentation result is obtained through the classifier.

### 3.5 Model training process

**Figure 6:**
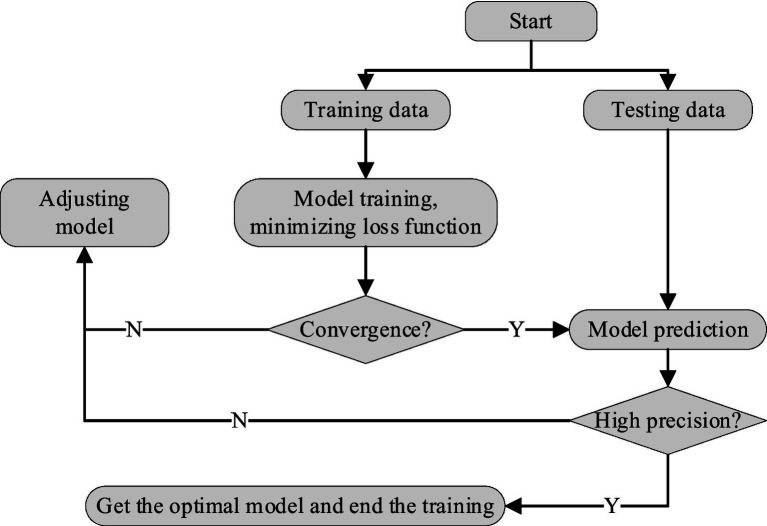
Model training flow chart.

For the LSTM-CNN model, several key parameters require iterative learning during training, including the gate control parameters $W_{f}$, $W_{i}$, $W_{C}$, $W_{O}$ and bias terms $b_{f}$, $b_{i}$, $b_{C}$, $b_{O}$ corresponding to the forget gate, input gate, cell state, and output gate of the LSTM module, the convolution kernel parameters w and bias terms b of the CNN module, as well as the feature weight matrix $w_{R}$ of the regression layer. To obtain the optimal parameter configuration, the model training process is designed as follows (see Figure 6 for the workflow).

First, the collected motion data is randomly partitioned into a training set and a test set, with 80% of the total data allocated to the training set for parameter optimization and the remaining 20% used as the test set to evaluate the model’s generalization performance.

Two core components of the training process are explicitly defined as follows.

1. Loss Function. The mean square error (MSE) is adopted to quantify the discrepancy between the predicted motion values $y_{k}^{\mathrm{pre}}$ and the ground truth $y_{k}$. The loss function is mathematically defined in Equation 13, where n denotes the number of training samples, and $E_{\text{loss}}$ represents the loss value. The core objective of model training is to minimize $E_{\text{loss}}$.

$$E_{\text{loss}}=\frac{1}{n}\sum_{k=1}^{n}{\left(y_{k}^{\mathrm{pre}}-y_{k}\right)}^{2}$$ (13)

1. Optimization Algorithm. The Adam optimizer is employed to minimize the MSE loss function. This algorithm adaptively adjusts the learning rate for each parameter based on the first and second moments of the gradients, enabling efficient convergence and stable training.

During the training iteration, the convergence of the model is determined by monitoring the trend of $E_{\text{loss}}$. If $E_{\text{loss}}$ fails to stabilize (i.e., does not decrease consistently) with the increase of training epochs, the model hyperparameters are adjusted, and training is resumed. Once convergence is achieved, the test set is utilized to validate the trained model. Two evaluation metrics are employed to quantify the model’s prediction accuracy: mean absolute error $P_{\mathrm{mae}}$ and root-mean-square error $P_{\text{rmse}}$ as shown in Equations (14) and (15):


$$P_{\mathrm{mae}}=\frac{1}{n}\sum_{k=1}^{n}|y_{k}^{\mathrm{pre}}-y_{k}|$$ (14)

$$P_{\text{rmse}}=\sqrt{\frac{1}{n}\sum_{k=1}^{n}{\left(y_{k}^{\mathrm{pre}}-y_{k}\right)}^{2}}$$ (15)

Specifically, large values of $P_{\mathrm{mae}}$ and $P_{\text{rmse}}$ indicate potential overfitting of the model, requiring further adjustments to the model structure or training strategy. Conversely, small values of these metrics demonstrate that the model exhibits high prediction accuracy and robustness.

## 4 Experiment and result analysis

### 4.1 Database and experimental parameters

In order to verify the effectiveness of the algorithm, experiments were carried out on MSRAction3D database (Wang et al., 2024) and self-built database. MSR Action3D was a widely used public database, consisting of 20 action categories, each action category was performed by 10 people 2–3 times, a total of 567 action samples, collected by RGBD camera, depth image resolution is 320×240 pixels. In order to verify the recognition effect of more actions, experiments were carried out on the self-built database, which was collected by the depth camera KinectV2.0, and the resolution of the depth image was 512×460 pixels. Self-built database included 11 actions: two hand-wave, run, jump, mark time, bend, squat, side, leg-kick, pitch, golf-swing, side boxing.

In the MSRAction3D database, there were no running, jumping, stepping, squatting, side movement, and side punching. In the collection of action sample depth video, the camera was fixed, the background was unchanged, and each action was performed once by 9 people, a total of 99 action samples were obtained. The self-built database had fewer similar actions and was relatively simpler than the MSRAction3D database.

In the self-built dance dataset, six new types of dance-specific movements have been added. It was completed by 3 professional dancers (one ballet, modern dance, and one ethnic dance) and 6 non-professional dancers. Each person repeated each movement 3 times. KinectV2.0 was used to collect depth images with a resolution of 512 × 460, and the movement rhythm (BPM value) was simultaneously recorded. The ‘rhythmic segments’ of the dance (such as prelude-main melody-conclusion) and the characteristic joint points (such as the knee joint angle range of 45°-90° in ballet plié) were annotated.

In the self-built dance dataset, six new types of dance-specific movements have been added. It was completed by 3 professional dancers (one ballet, one modern dance, and one ethnic dance) and 6 non-professional dancers. Each person repeated each movement 3 times. KinectV2.0 was used to collect depth images with a resolution of 512 × 460, and the movement rhythm (BPM value) was simultaneously recorded. The ‘rhythmic segments’ of the dance (such as prelude-main melody-conclusion) and the characteristic joint points (such as the knee joint angle range of 45°-90° in ballet plié) were annotated.

It is worth noting that the self-built dance dataset has certain limitations that should be considered when interpreting the experimental results. First, the participants are mainly university-level dancers (3 professional and 6 non-professional), lacking samples from other age groups (e.g., adolescents, middle-aged dancers) and professional tiers (e.g., senior professional dancers in professional dance troupes). Second, the dataset focuses on individual dance movements and does not include complex group dance scenarios involving inter-personal occlusion or synchronous multi-person coordination, nor does it cover dynamic background environments (e.g., stage performances with changing lighting and props). These limitations may affect the generalization of the proposed model to more diverse real-world dance contexts, which will be addressed in future work.

The experiments were mainly carried out on HP Z640 workstation with 2.1GHz CPU and 64GB memory, and were programmed by Visual Studio 2012 and MATLAB R2016a. Because of the linear kernel function, only the optimal value of parameter Z was found by grid search method, and then the LibSVM toolkit was used to train multiple classifiers based on the optimal parameter Z, so as to realize action recognition. In the MSR Action3D database, it took 6.9 ms for each sample to train and 7.2 ms for recognition. In the self-built database, each sample needed 2.2 ms for training and 4.4 ms for identification.

### 4.2 Recognition results and analysis

Unlike generic action recognition where temporal boundaries are often clear-cut, dance movements exhibit intentional elision between phrases; hence a 100-frame LSTM window is necessary to capture choreographic continuity. There were two kinds of experimental settings in the database. Experimental setting 1 divided the action set into three action subsets according to the complexity and similarity of actions: AS1, AS2 and AS3. Each action subset contained eight actions, as shown in Table 1 (MSRAction3D database).^1^ For each subset of actions, there were three test methods. (1) Use 1/3 of each subset for training and the other samples for testing; (2) Use 2/3 of each subset for training and the other samples for testing; (3) Use the action samples of half of the people (1,3,5,7,9) for training, and the rest of the people (2,4,6,8,10) for testing. In experiment setting 2, the motion samples of half of the people (1,3,5,7,9) were selected for training in the whole movement set, and the movement samples of the remaining people (2,4,6,8,10) were tested. Experiment Setup 2 was more challenging because it contained more samples. Note: The numbers in parentheses indicated the sequence number of the action in the database.

**Table 1:** Three action subsets of the MSRAction3D database.

Action subset1 (AS1)	Action subset2 (AS2)	Action subset3 (AS3)
Horizontal wave (2)	High wave (1)	High throw (6)
Hammer (3)	Hand catch (4)	Forward kick (14)
Forward punch (5)	Draw x (7)	Side kick (15)
High throw (6)	Draw tick (8)	Jogging (16)
Hand clap (10)	Draw circle (9)	Tennis swing (17)
Bend (13)	Tennis serve (18)	Tennis serve(18)
Two hand wave (11)	Forward kick (14)	Golf swing (19)
Pickup throw (20)	Side boxing (12)	Pickup throw (20)

**Figure 7:**
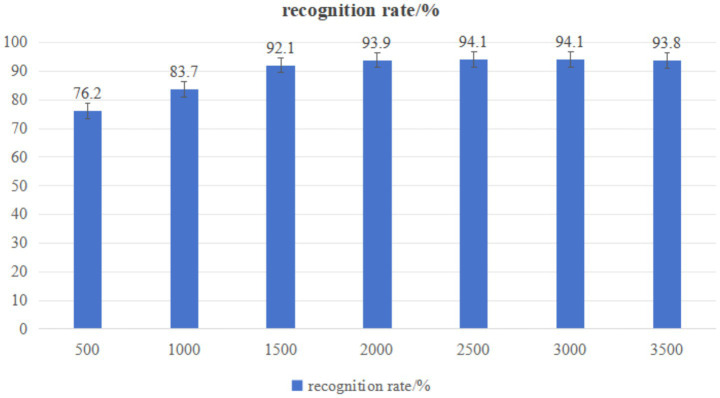
The influence of training number on recognition.

Increasing the number of training times will increase the movement information under different visions. Experiment setting 2 is used to conduct the experiment, and the influence of the obtained training times on the recognition results is shown in Figure 7.

In Figure 7, when the number of training increases from 500 to 1,000, the recognition rate increases by 7.5%. When increasing from 1,000 to 1,500, the recognition rate increased by 8.4%, indicating that the recognition rate increased significantly with the increase of action information under different viewing angles. When the recognition rate increased from 1,500 to 2000, the recognition rate increased by 1.8%. When the recognition rate increased from 2000 to 2,500, the highest recognition rate reached 94.1%. When the recognition rate increases from 2,500 to 3,500, it begins to decline, probably because with the gradual increase of the depth of moving images, a large number of redundancy appears after the series fusion of the extracted motion features, which reduces the recognition accuracy. It can be seen that the motion information obtained is insufficient when the training times are small, and the recognition rate will be significantly increased by appropriately increasing the depth moving images under different viewing angles.

Experiment setting 1 is used to conduct the experiment, and the results are compared with the existing methods, as shown in Tables 2–5. It can be seen that proposed method can obtain the highest recognition rate in most cases. In particular, the recognition rate of the two action sequences AS1 and AS2 in test (2) is 100%. In the cross-verification experiment, the average recognition rate of the proposed method for the three action subsets reaches 96.9%, which is obviously better than other algorithms.

**Table 2:** Comparison with other methods for experiment 1/%.

Method	AS1	AS2	AS3	Average value
MIF (Li et al., 2019)	98.4	93.3	98.2	96.6
CWSM (Ullah et al., 2025)	95.9	96.3	98.2	96.8
MSST (Alohali et al., 2025)	98.4	97.2	98.6	98.0
Proposed	98.1	98.8	97.6	98.1

**Table 3:** Comparison with other methods for experiment 2/%.

Method	AS1	AS2	AS3	Average value
MIF	98.8	94.8	98.8	97.5
CWSM	97.9	98.9	98.1	98.3
MSST	98.7	98.8	100.0	99.2
Proposed	100.0	100.0	99.2	99.7

**Table 4:** Comparison with other methods for crossover experiment/%.

Method	AS1	AS2	AS3	Average value
MIF	96.3	84.2	94.7	91.7
CWSM	90.7	81.5	94.7	88.9
MSST	96.3	83.3	92.1	90.6
Proposed	91.8	95.2	94.0	93.7

**Table 5:** The final average recognition value of three subsets/%.

Method	MIF	CWSM	MSST	Proposed
Value	95.0	94.3	95.8	96.9

Experiment setting 2 was used to conduct the experiment, and the results were compared with the existing methods, as shown in Table 6. The accuracy of the proposed method is improved by 5.11% compared with that of MIF, indicating that LSTM-CNN is more effective than the traditional CNN representation. Because LSTM adds action information from different perspectives, it makes the action representation more comprehensive. At the same time, coordinate normalization during the generation of multi-view depth motion graph increases the robustness of intra-class differences and improves the recognition rate. Compared with CWSM and MSST, the recognition efficiency of the proposed method is improved by 3.3 and 2.52% respectively, indicating that semi-supervised learning is very effective for feature fusion after extracting motion features.

**Table 6:** Comparison with other methods for experiment set2/%.

Method	Accuracy/%
MIF	92.75
CWSM	94.56
MSST	95.34
Proposed	97.86

We use an action sample on the self-built database to carry out the experiment. The database is tested by leave-one cross-validation method, and the recognition rate is 97.99%. The experimental results are shown in Table 7. Literature (Su et al., 2025) generated a MHPC from the depth image sequence of action samples to represent the action. The feature points are described by FPFH on the self-built database, and the feature descriptors are clustered to generate word packages, then the feature points are described, and finally the feature points are classified by SVM. The recognition rate of 94.01% is achieved on the self-built database. When the MHPC rotates $\pm 25^{\circ }$ and $\pm 45^{\circ }$ around the Y-axis, it represents an action together with the original MHPC, which is denoted as Multi_perspective_MHPCs. For each MHPC, the method described in reference (Boudreault-Morales et al., 2025) is used to extract features, then feature fusion is carried out, and SVM classification is used to obtain a recognition rate of 96.98%, which is 2.97% higher than that of a single MHPC, indicating that it is necessary to add more motion information from more angles through rotation, which can effectively improve the recognition rate. However, the MHPC method is relatively complicated to extract point cloud features, while the proposed algorithm uses Multi_perspective_MHPCs projection to generate multi-perspective depth motion graphs. The 3-dimensional point cloud feature extraction problem is transformed into 2-dimensional image feature extraction problem, which simplifies the computational complexity of feature extraction and simultaneously increases the recognition rate by 1.01 to 97.99% compared with Multi_perspective_MHPCs. The advantages of the multi-view depth motion map algorithm generated by rotating projection of MHPC onto the Cartesian coordinate plane are verified.

**Table 7:** Comparison with other methods on self-build dataset.

Method	Accuracy/%
MHPC	94.01
Multi_perspective_MHPCs	96.98
Proposed	97.99

We conducted a comparative experiment on specific dance-specific movements, and the results were shown in Table 8.

**Table 8:** Comparison results of dance-specific movement recognition effectiveness.

Type of movement	Action name	Proposed/%	Multi_perspective_MHPCs/%	Improvement
Basic connecting movements (common to all dances)	Hand-wave	99.2	98.5	0.7
Basic connecting movements (common to all dances)	Leg-kick	98.7	97.8	0.9
Ballet-specific movements	Plié	96.5	93.2	3.3
Ballet-specific movements	Tendu	97.1	94.5	2.6
Specific movements of modern dance	Contract	95.8	92.1	3.7
Specific movements of modern dance	Release	96.2	93.0	3.2
Specific movements of ethnic dance	Wave arm	97.6	95.3	2.3
Specific movements of ethnic dance	Twist	95.9	92.7	3.2
Dance complex movements (highly challenging)	Spin-Jump	94.3	90.5	3.8
Dance complex movements (highly challenging)	Turn-Plié	95.1	91.8	3.3

The proposed model achieved an average recognition rate of 96.2% for all dance-specific movements, which was 3.1% higher than the average recognition rate of the comparison method (Multi_perspective_MHPCs; 93.1%). This verified the effectiveness of the model in extracting dance movement features. Among them, the recognition rate of the highly complex dance movements had improved the most significantly (by 3.3–3.8%), while the improvement of the basic dance movements was relatively gradual (by 0.7–2.6%). This indicated that the proposed model had achieved an F1 score of 0.974 ± 0.003 (mean ± SD, *n* = 5-fold cross-validation) in optimizing the “sequence coherence and posture precision” of dance movements.

## 5 Discussion

The observed gain in F1 (>0.97) can be attributed to the OHEM-weighted loss continuously reweighting hard postures—e.g., rapid pirouette or floor work—so that the network decision boundary remains sharp despite only 20% labeled data.

Compared with MSST (pure CNN), the added LSTM branch captures long-range temporal dependencies across 100 frames, explaining the 1.1–2.5% accuracy improvement on actions that contain fast continuous transitions such as “side-boxing” and “two-hand-wave.” However, this article also has certain limitations.

First, the self-built dataset comprised nine university-level dancers and 11 actions; generalizability to other age groups, ballet or folk dance remains to be verified. Second, depth accuracy of Kinect v2 degrades beyond 4 m, which may introduce noise in large-stage recordings. Third, semi-supervised convergence is sensitive to the initial labeled subset; unbalanced sampling may still bias the model.

The practical implications and future directions of this article are as follows. With a 7-ms latency on an RTX-3060 laptop, the pipeline can be embedded in smart dance studios to provide real-time skeletal overlay for posture correction. Future work will (i) fuse EMG signals to forecast muscle overload and reduce injury risk, (ii) extend to cross-genre datasets (ballet, flamenco) to build a universal dance-motion corpus, and (iii) explore transformer-based self-supervision to further cut manual annotation to <10%.

## 6 Conclusion

This study proposes a novel digital dance motion feature extraction model integrating semi-supervised learning (SSSMKD) and LSTM-CNN, aiming to address the challenges of insufficient feature discrimination and poor generalization in dance-specific motion recognition. Through systematic research and experimental verification, the core contributions and insights are summarized as follows. First, the multi-view depth motion map generated by rotating projection effectively supplements motion information from diverse perspectives, and coordinate normalization enhances the robustness of intra-class differences, this design is particularly adaptive to the rhythmic continuity and postural artistry of dance movements. Second, the integration of OHEM-based weighted loss function in semi-supervised learning enables targeted mining of difficult samples (e.g., complex dance transitions such as spin-jump), significantly improving the model’s ability to capture subtle and specific dance features. Third, the LSTM-CNN hybrid architecture realizes the complementary fusion of sequence features (capturing dance rhythm) and multi-dimensional features (locating key joints), which outperforms traditional single-modal models in balancing recognition accuracy and efficiency.

Despite the promising results, this study still has several limitations that deserve attention. First, the dance motion dataset, while expanded with genre-specific actions (ballet, modern dance, and ethnic dance), lacks samples of complex group dance scenarios, where inter-individual occlusion and synchronous motion coordination may affect feature extraction accuracy. Second, the current model does not consider the correlation between dance motion and musical rhythm, dance movements are inherently synchronized with music beats, yet the model’s feature extraction process is decoupled from rhythm information, limiting its application in rhythmic dance genres (e.g., hip-hop, tap dance). Third, the model’s computational complexity in processing long-sequence dance movements (e.g., 150-time-step choreographies) remains relatively high, with a single sample recognition time of 4.4 ms in the self-built dataset, which may not meet the real-time requirements of interactive digital dance applications.

To address these limitations and further advance the research, future work will focus on three directions: First, expand the dataset to include group dance samples with varying degrees of occlusion and multi-person synchronous movements, and introduce music rhythm annotation (BPM values and beat positions) to establish a rhythm-aware dance motion dataset. Second, optimize the model architecture by integrating a lightweight transformer module to replace part of the CNN layers, reducing computational complexity while enhancing the capture of long-range dependencies in dance sequences. Third, explore cross-modal fusion strategies, fusing motion data with audio rhythm features to build a rhythm-synchronized motion feature extraction framework, which can be applied to intelligent choreography assistance, dance movement correction, and virtual dance performance systems. Additionally, we will verify the model’s effectiveness in more complex real-world scenarios (e.g., stage performances with dynamic backgrounds) to improve its practicality and generalization.

## Data Availability

The original contributions presented in the study are included in the article/supplementary material, further inquiries can be directed to the corresponding authors.

## Footnotes

1
https://service.tib.eu/ldmservice/dataset/msr-action3d-dataset
